# A Putative P-Type ATPase Required for Virulence and Resistance to Haem Toxicity in *Listeria monocytogenes*

**DOI:** 10.1371/journal.pone.0030928

**Published:** 2012-02-21

**Authors:** Heather P. McLaughlin, Qiaobin Xiao, Rosemarie B. Rea, Hualiang Pi, Pat G. Casey, Trevor Darby, Alain Charbit, Roy D. Sleator, Susan A. Joyce, Richard E. Cowart, Colin Hill, Phillip E. Klebba, Cormac G. M. Gahan

**Affiliations:** 1 Alimentary Pharmabiotic Centre, Department of Microbiology, University College Cork, Cork, Ireland; 2 School of Pharmacy, University College Cork, Cork, Ireland; 3 Department of Chemistry and Biochemistry, University of Oklahoma, Norman, Oklahoma, United States of America; 4 Department of Biological Sciences, Cork Institute of Technology, Cork, Ireland; 5 Université Paris Descartes, Faculté de Médecine Necker-Enfants Malades, Paris, France; 6 INSERM, U1002, Unité de Pathogénie des Infections Systémiques, Paris, France; 7 Division of Biological Science, Department of Natural and Applied Sciences, University of Dubuque, Dubuque, Iowa, United States of America; Vrije Universiteit Brussel, Belgium

## Abstract

Regulation of iron homeostasis in many pathogens is principally mediated by the ferric uptake regulator, Fur. Since acquisition of iron from the host is essential for the intracellular pathogen *Listeria monocytogenes*, we predicted the existence of Fur-regulated systems that support infection. We examined the contribution of nine Fur-regulated loci to the pathogenicity of *L. monocytogenes* in a murine model of infection. While mutating the majority of the genes failed to affect virulence, three mutants exhibited a significantly compromised virulence potential. Most striking was the role of the membrane protein we designate FrvA (Fur regulated virulence factor A; encoded by *frvA* [*lmo0641*]), which is absolutely required for the systemic phase of infection in mice and also for virulence in an alternative infection model, the Wax Moth *Galleria mellonella*. Further analysis of the *ΔfrvA* mutant revealed poor growth in iron deficient media and inhibition of growth by micromolar concentrations of haem or haemoglobin, a phenotype which may contribute to the attenuated growth of this mutant during infection. Uptake studies indicated that the *ΔfrvA* mutant is unaffected in the uptake of ferric citrate but demonstrates a significant increase in uptake of haem and haemin. The data suggest a potential role for FrvA as a haem exporter that functions, at least in part, to protect the cell against the potential toxicity of free haem.

## Introduction

Iron is indispensable for the growth of most bacteria, serving as a cofactor for enzymes involved in essential metabolic pathways such as glycolysis, DNA synthesis, energy generation, and detoxification of oxygen radicals [1], [2]. The correlation between iron acquisition and bacterial virulence has been well documented [3], [4], [5] and the absolute requirement for this metal for both host metabolism and bacterial growth results in significant competition for iron in the host [6]. Following bacterial infection host responses are evoked which sequester iron, making it relatively unavailable for bacterial metabolism [7].

In the Gram positive intracellular pathogen *Listeria monocytogenes*, iron deficient environments have been shown to upregulate the expression of the principal virulence regulator PrfA and significantly increase the production of the haemolysin Listeriolysin O promoting phagosomal escape, and the actin polymerisation protein ActA which plays a role in cell-to-cell spread [8], [9], [10]. It has been hypothesized that the requirement for iron has played a part in driving the evolution of an intracellular life-cycle for *L. monocytogenes* as the bacterium can utilize the iron-saturated protein ferritin stored in the cytosol of host cells (as reviewed by McLaughlin *et al*. [11]).

As iron-limiting conditions can be encountered in both the natural environment and during host infection, free-living pathogenic bacteria such as *L. monocytogenes* have evolved mechanisms to acquire iron from a variety of sources. Iron acquisition is mediated by a number of distinct systems that have been characterized in *L. monocytogenes*: a citrate inducible receptor for the uptake of ferric citrate, utilization of exogenous siderophores, catechol siderophore-like molecules, and catecholamine complexes, and iron acquisition via a cell-surface transferrin-binding protein [12]. A comprehensive analysis of the iron acquisition systems in *L. monocytogenes* identified a variety of iron sources which can be used for growth, including eukaryotic iron-binding proteins (haemoglobin, ferritin, transferrin and lactoferrin), ferric siderophores (enterobactin and corynebactin) and iron complexes of hydroxymates (ferrichrome, ferrichrome A, and ferrioxamine B) [2]. In addition, the same study also identified two genetic loci responsible for the uptake of ferric hydroxymates and haemin/haemoglobin. Deletions in *fhuD* or *fhuC* strongly reduced ferrichrome uptake and a deletion in *hupC* eliminated uptake of haemin and haemoglobin and resulted in decreased virulence potential [2]. However, it is clear that many other loci putatively involved in iron homeostasis in *L. monocytogenes* remain to be characterized by functional genetics approaches [13], [14].

Maintaining a balanced acquisition of iron from the external environment is essential for bacterial survival. Whilst pathogens must compete for iron during infection excess intracellular iron can lead to the generation of toxic hydroxyl radicals via the Fenton reaction. Iron homeostasis in most bacteria, including *L. monocytogenes*, is controlled by the regulatory protein Fur (ferric uptake regulator) or a functional equivalent [15]. In the presence of sufficient levels of iron, Fur acts as a repressor whereby an iron-Fur complex prevents gene transcription by binding to specific Fur-box sequences upstream of the start codon of target genes [16].

Recently Ledala *et al*. [17] used DNA microarray analysis to examine gene expression in a Fur mutant and identified Fur-regulated genes in *L. monocytogenes*, including genes encoding iron transporters and proteins involved in iron storage. In this study, we undertook an independent genome-wide search to identify putative Fur-box consensus sequences in the genome of *L. monocytogenes*. This approach identified a number of candidate Fur-regulated loci, including some (such as *lmo0641*) that were not identified previously through microarray analysis [17]. We undertook a systematic functional analysis of selected Fur-regulated loci by creating plasmid-insertion mutants and subsequently testing these for virulence potential in the murine model. This led to the identification of Fur-regulated virulence factor A, FrvA (encoded by *frvA*/*lmo0641*), a novel Fur regulated virulence factor which is absolutely required for growth of *L. monocytogenes* under restricted iron conditions and for systemic infection. We carried out iron uptake studies on the *frvA* mutant and determined that the mutant demonstrates a significant increase in uptake of haem and is also sensitive to elevated haem concentrations. Sensitivity to haem toxicity may account for the significant attenuation of virulence during the systemic phase of infection in the murine infection model.

## Results and Discussion

### *In silico* identification of putative Fur regulated genes

Fur has been identified as a major regulator of iron homeostasis in numerous Gram-positive and Gram-negative bacteria [16], [18], [19]. Regulation of iron uptake is particularly important during infection as pathogens must scavenge iron from sources in the host organism. Indeed, deregulation of iron uptake through elimination of Fur has been shown to significantly impact upon virulence potential in a number of pathogenic bacteria, including *L. monocytogenes* [20], [21]. Surprisingly, recent approaches to identify novel *in vivo*-induced genes in *L. monocytogenes* (such as microarray and IVET approaches) have failed to identify the key inducible systems for iron-uptake during infection [22], [23], [24]. In addition, signature tagged mutagenesis approaches have also failed to identify the mechanisms of intracellular iron uptake in this pathogen [25]. We therefore employed a systematic functional genetic analysis of selected Fur-regulated genes and identified a locus (*lmo0641*, now designated *frvA*) that is absolutely required for the systemic phase of *L. monocytogenes* infection.

**Figure 1:**
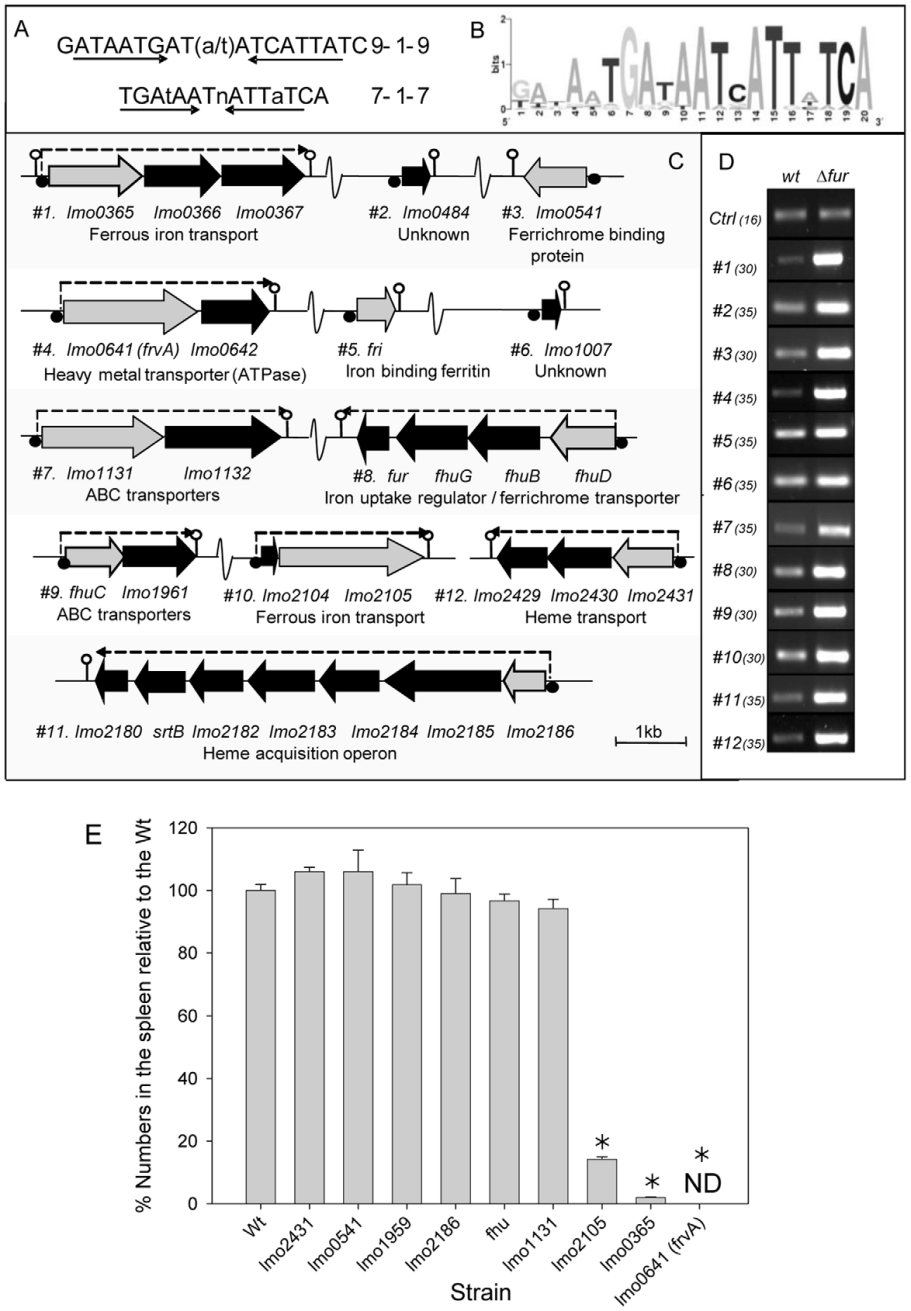
Identification and role in virulence of Fur-regulated gene systems. (A) The classical Fur box is represented as a 19 bp sequence. Recent studies have suggested that a more accurate representation of the Fur box is that of a 7-1-7 motif. The 19 bp sequence was used to search the *Listeria monocytogenes* EGDe genome sequence (Listilist). (B) Identified sequences were aligned and a graphical display of the results was generated using the web based programme sequence logo (17). (C) Genetic organisation of 29 putative Fur regulated genes (black/gray) at 12 chromosomal loci. All genes are drawn approximately to scale using the *L. monocytogenes* EGDe genome sequence data. Lmo numbers refer to the National Centre for Biotechnology Information annotation scheme. Fur boxes are represented by black circles. Gray genes indicate those disrupted in EGDe in the course of this study. Lollipops are used to illustrate putative stem loop terminator regions. (D) RT-PCR analysis was used to confirm Fur regulation of all identified genes and to give an indication of the increase in expression levels. Control primers were used to ensure that template cDNAs were of equal concentration. Samples were removed at various cycles of PCR (cycle number in brackets) and visualised on agarose gels. A repeat experiment demonstrated similar results. Results were also verified through real-time PCR analysis. (E) In vivo survival of disruption mutants in Fur-regulated loci in the murine infection model. Mice were injected i.p. with either the wild-type or mutants and the number of bacteria recovered from the spleen was determined three days post-inoculation. Error bars represent the standard deviations from the mean (n = 4). * indicates means are significantly different to the wild-type (P<0.05). ND, not detected.

Ledala and coworkers have recently utilised microarray analysis to identify members of the Fur regulon in *L. monocytogenes* [17]. We concurrently used the classical 19 bp Fur-binding motif defined in *B. subtilis* [26] (**Figure 1A**) to mine the *L. monocytogenes* EGDe genome for similar motif sequences. We used two primary criteria to limit the number of sequences identified. Firstly, the identified sequence should be within 350 bp of an annotated start codon and secondly, a match at 16 or more of the 19 positions was required. Anything less than 16/19 was not considered unless the annotated ORF was deemed to have a likely role in iron acquisition based on bioinformatic analysis. This approach identified a subset of the Fur-regulated loci determined through microarray analysis [17]. However, we also identified Fur-regulated loci at *lmo2431* (previously identified as a potential Fur-regulated locus by Jin *et al*. [2] and *lmo0641* (the subject of this study) which were not detected using the cut-off criteria employed by Ledala *et al.* [17]. Another locus (*lmo0484*) was identified here which is adjacent to a gene (*lmo0485*) identified using microarrays and therefore may form part of an operon.

The loci identified as containing Fur-binding motifs are represented in **Figure 1**. In each case, where the Fur box was upstream of a putative operon, RT-PCR confirmed co-transcription of all the genes in the operon (data not shown). The Fur boxes were aligned and a graphical display of the results was generated using ‘sequence logo’ which generates a consensus for *Listeria* that is identical to that in *Bacillus* (**Figure 1B**) [27]. Fur regulation was confirmed through RT-PCR analysis of representative genes in both wild-type *L. monocytogenes* and a Δ*fur* mutant. The results validated the microarray data described previously [17] and also confirmed that *lmo2431* and *lmo0641* are regulated by Fur.

### Virulence analysis of plasmid insertion mutants

We created mutants using the pORI19 integration strategy as this method is relatively rapid, results in stable mutations and lends itself to analysis of a large number of loci in a reasonable timeframe [20], [28]. Two of the identified loci (*lmo1007* and *lmo0484*) consisted of a single small (<500 nt) gene and were considered too small for plasmid disruption and were not analysed here. Mutation of *fri* has been described elsewhere [29], [30], [31]. Where the Fur box was upstream of an operon we chose the first open-reading frame for plasmid disruption as this would increase the likelihood of causing pleiotropic effects on co-transcribed downstream genes. Plasmid disruptions at the correct locations were confirmed by PCR, using a primer based on the EDGe chromosome and one based on the plasmid. The absence of the *repA* gene in mutant strains selects against excision and extrachromosomal maintenance of the integration plasmid, ensuring stable integrants for subsequent analysis (see experimental procedures for details). mRNA was extracted from each of the mutants and RT-PCR analysis confirmed that plasmid disruption of the target gene was associated with the complete elimination of expression from each locus with the exception of the *lmo2431* mutant (a locus previously analysed by Jin *et al.* [2]) in which gene expression was greatly reduced (data not shown).

In this initial screen, three of the mutants in Fur-regulated loci exhibited a significant reduction in virulence potential relative to the wild type (P<0.05) (**Figure 1E**). The most significantly affected mutant in this screen was pORI19::*frvA*.

### The Fur-regulated virulence (*frvA*) locus is required for effective infection

**Figure 2:**
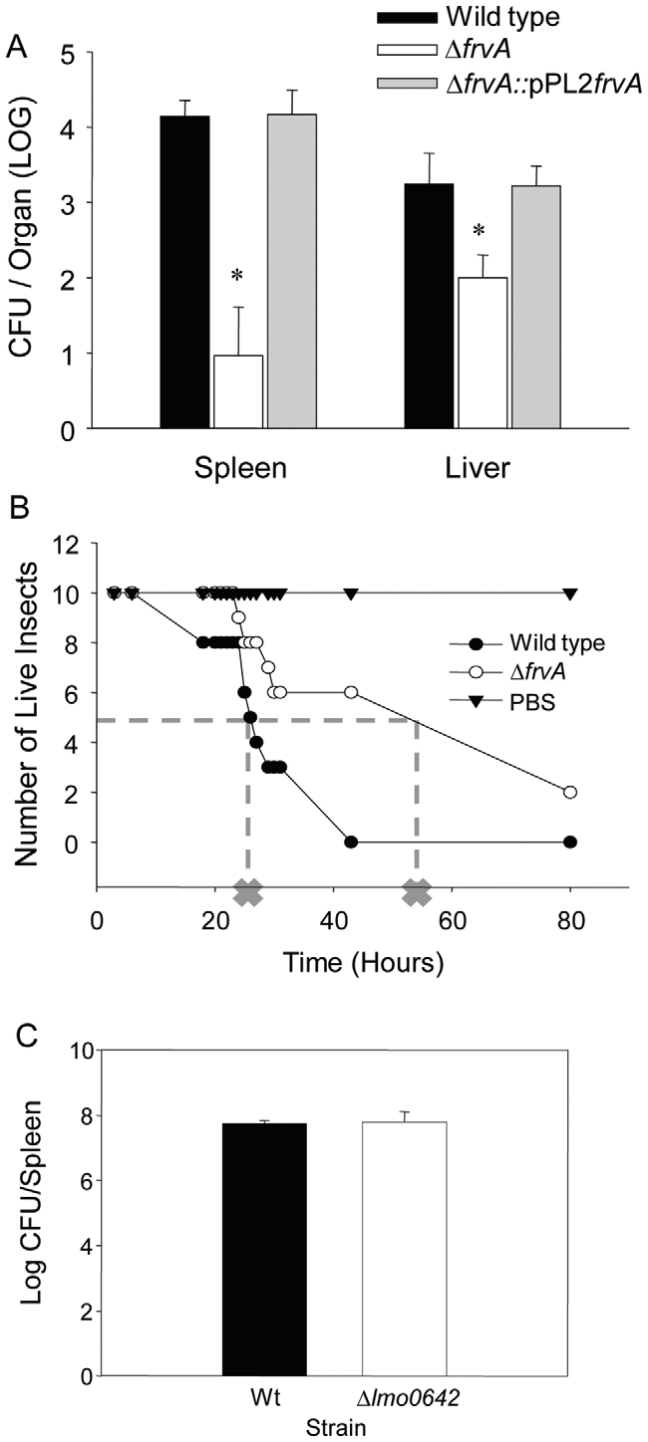
Pathogenesis of strains in murine and Wax Moth models of infection. (A) CFUs of the Δ*frvA* and Δ*frvA* complemented strain enumerated from livers and spleens three days post infection. Error bars represent standard error of the mean and asterisks represent P<0.001 by the Student's t-test when compared to the wild-type and complement strains. (B) Pathogenesis of strains in the *Galleria mellonella* model of infection. Dotted line and cross indicates LT-50 (time in which 50% of insects had perished). (C) Pathogenesis of the Δ*lmo0642* mutant in the murine model of infection. Strains were inoculated into mice by the ip route and numbers were enumerated in the spleens at day three post-infection. Student t-test did not detect a significant difference between the wild-type and mutant strain.

To confirm an essential role for *frvA* in the virulence of *L. monocytogenes* two precise in-frame deletion mutants were created (see experimental procedures). An initial mutant was created through the deletion of the central region of the *lmo0641* gene, from residues 85–416 inclusive (mutant designated Δ*frvA*_[85–416]_). As toxicity has previously been associated with the generation of truncated membrane proteins through partial deletion [31] we also created a precise deletion mutant in which the entire open reading frame was deleted. This mutant was designated Δ*frvA*. Both mutants were complemented using the pPL2 plasmid to re-introduce a single copy of *frvA* (designated Δ*frvA*::pPL2*frvA* and Δ*frvA*_[85–416]_::pPL2*frvA*). Although growth of Δ*frvA* was unaffected in nutrient-rich media (BHI), this mutant was recovered at significantly lower levels (three-log reduction) from the spleens of infected mice on day three post-infection when compared to the wild-type. Numbers recovered from the liver at three days post inoculation indicated a lesser, but still significant reduction in levels as compared to the wild-type. The reintroduction of *frvA* fully restored the virulence potential **(** **Figure 2A**). These data definitively establish a critical role for *frvA* in *L. monocytogenes* virulence potential and pathogenesis. Notably, Δ*frvA*_[85–416]_) also demonstrated a dramatic reduction in virulence potential in the murine model (**Figure S1**).

Larvae of the Wax Moth (*Galleria mellonella*) have recently been utilized as an alternative pathogenicity model for *L. monocytogenes* [32], [33]. Here we also analysed the virulence potential of *ΔfrvA* using this model system (**Figure 2B**). While no deaths were observed over time for the insects that received PBS, a significant difference was seen between insects receiving the wild-type and the *ΔfrvA* mutant. The LT-50 for insects infected with the wild-type strain was 26 hours while over 50% of the *ΔfrvA*-infected insects were still alive after 43 hours. The significance of iron acquisition to the virulence of bacterial pathogens has previously been investigated in this insect model. Work by Daou *et al*. [34] demonstrates a role for IlsA, a surface protein in *Bacillus cereus* that binds haemoglobin and ferritin, to pathogenesis in the *G. mellonella* host. In order to determine the possible influence of the downstream gene *lmo0642* in murine virulence of *L. monocytogenes* we created an in-frame mutation in this locus. Interestingly, this gene is apparently not required for pathogenesis. Deletion of *lmo0642* failed to affect the ability of *L. monocytogenes* to reach either the liver or spleen in mice infected by the intraperitoneal (i.p.) route (**Figure 2C**) or to grow intracellularly (data not shown).

*ΔfrvA* was compared to the wild-type and complemented strains for their ability to replicate within J774 macrophage cells (**Figure S2**). After one hour *ΔfrvA* displayed no significant difference in invasion of J774 cells when compared to the wild-type or complement strains. Subsequent readings taken after three, five and seven hours represent intracellular survival and multiplication of these strains within the cell line. Similarly, no significant difference was observed in the ability of *ΔfrvA* to survive inside J774 macrophages over time, as all three strains displayed growth of approximately one log after 7 hours.

### Bioinformatic analysis of *frvA*

FrvA is a putative transmembrane protein consisting of six predicted transmembrane regions (SOSUI) and is annotated as being similar to a heavy metal-transporting ATPase (http://genolist.pasteur.fr/ListiList/). The closest non-listerial homologues reside in *Bacillus* spp. A predicted heavy metal-transporting ATPase in *B. megaterium* was found to share 56% identity and 72% similarity (over a query coverage of 621/626 amino acids) with FrvA. A predicted cadmium-transporting ATPase in *B. halodurans* C-125 also shared close homology with 55% identity and 72% positives (over 618/626 amino acids) (NCBI Blast). Three conserved domains were identified in FrvA using the Conserved Domain Search from NCBI including a P-type ATPase superfamily, a haloacid dehalogenase-like (HAD) hydrolase, and a cation transport ATPase. In addition, FrvA was found to contain several classic P-type ATPase motifs such as the phosphorylation motif D^321^KTGTLT and the hinge region motif G^518^DGIND. Similar to other type I heavy metal-transporting ATPases such as YkvW in *Bacillus subtilis* and CtpA, a P-type ATPase involved in copper homeostasis in *L. monocytogens*, FrvA also possesses both an M4 motif S^277^PC and an HP motif, S^358^LHPLA, respectively [35].

Lmo0642, the product of the downstream gene, is also predicted to be localized to the bacterial membrane (PSORT) and also has 6 transmembrane regions (SOSUI). No conserved domains were identified (NCBI) and its closest homolog is a hypothetical protein (EF0716) from *Enterococcus faecalis* V583 (NCBI Blast).

### Regulation of *frvA* by Fur

qRT-PCR analysis of the wild-type *L. monocytogenes* EGDe strain and a *Δfur* mutant confirmed that *lmo0641* is under the negative regulation of Fur. Using the 2^−ΔΔCt^ method to calculate the relative changes in gene expression, *lmo0641* was shown to be up-regulated 93-fold in *Δfur* compared to the wild-type. Transcription of *frvA* was also found to under the control of PerR, a Fur homolog which functions as an Fe(II)-dependent peroxide stress sensor and which regulates putative metal transport and storage functions [36]. In addition to the classical Fur box a putative PerR binding region was identified upstream of the annotated start codon of *frvA.* De-repression of *frvA* was seen in the absence of either regulator. However, no further impact was observed in a Δ*fur*Δ*perR* double mutant (data not shown). The significance of this dual regulation by Fur and PerR is unclear, but highlights some degree of interplay between these two regulators. It is interesting to note that *frvA* (*lmo0641*) was also previously determined to be regulated by PrfA, the master regulator of virulence gene expression in *L. monocytogenes* [37]. The locus is not preceded by a detectable PrfA binding motif but the authors noted the presence of a binding site recognized by Sigma B, the general stress response regulator. Taken together, the evidence suggests that the locus is a member of multiple regulatory networks, perhaps reflecting the importance of FrvA in *L. monocytogenes* niche adaptation.

### Δ*frvA* displays increased haemin uptake and elevated sensitivity to haem toxicity

**Figure 3:**
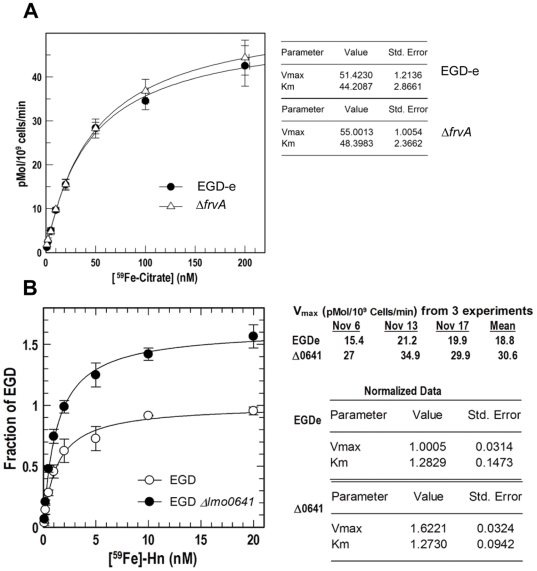
^59^Fe binding and uptake assays. Uptake affinity (K_m_ in nM) and velocity (V_max_ in pMol per 10^9^ cells per minute) by which the wild-type (open circles) and Δ*0641* (Δ*frvA*) (closed circles) strains transport [^59^Fe]-citrate (A) and [^59^Fe]-Hn (B) were assessed. Overall K_m_ and V_max_ of [^59^Fe] transport are listed in the tables on right-hand side. Data was plotted using the Enzyme Kinetics algorithm of Grafit 7 (Erithacus Ltd, West Sussex, UK) and represent the mean of independent experiments done in triplicate.

In an attempt to understand the virulence defect displayed by Δ*frvA* we carried out extensive physiological analysis of the mutant strain. A [^59^Fe]-citrate uptake assay indicated that the ability of Δ*frvA* to acquire ferric citrate was not impaired when compared to the wild-type *L. monocytogenes* strain (**Figure 3A**). Both strains transport ferric citrate with similar affinity (K_m_) and velocity (V_max_). Although Adams *et al*. [38] have reported that a citrate inducible iron uptake system exists in *L. monocytogenes* we demonstrate here that the FrvA system is not involved in the direct uptake of ferric citrate. The existence of an iron reductase has previously been suggested in *L. monocytogenes* based upon physiological data [39], [40] although this remains the subject of some debate [2]. We performed iron reductase assays but could find no significant difference between wild-type and mutant cells in ability to reduce iron in these assays, suggesting that this locus does not encode an iron reductase (see **Table S1**).

**Figure 4:**
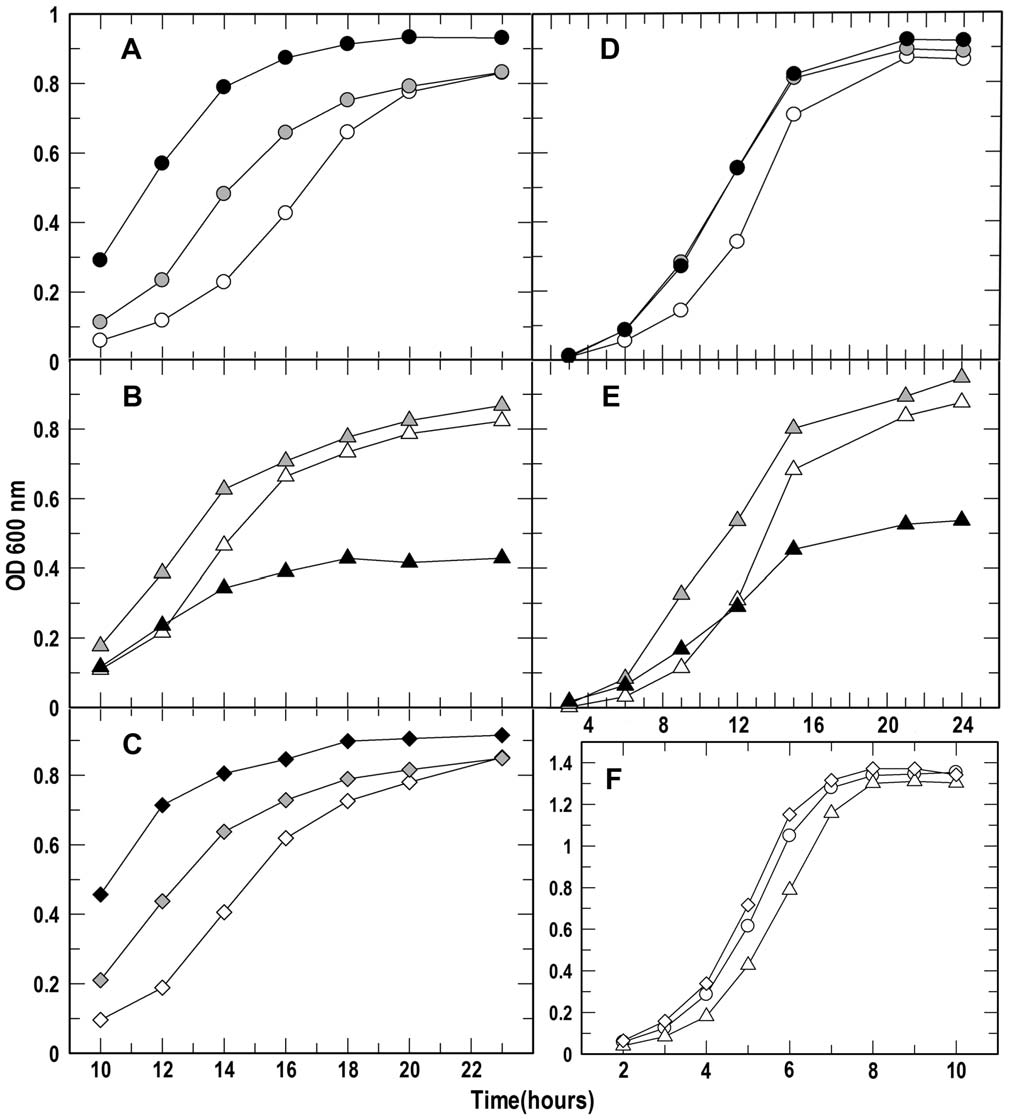
Bacterial growth. The rates and extent of bacterial growth (A: EGD-e; C: Δ*lmo0641*; E:Δ*lmo0641*/pPL2*lmo0641*) were determined in iron-restricted MOPS-L media supplemented with Hb (panels **A**–**C**; open, gray and black symbols represent addition of 0.0, 0.02 and 2 uM Hb, respectively) or Hn (**D**, **E**; open, gray and black symbols represent addition of 0.0, 0.2 and 2 uM Hn, respectively), and in BHI broth (**F**). The bacteria were cultured in BHI broth overnight. In **A–E** they were then subcultured in MOPS-L to stationary phase, and at t = 0 subcultured again at 1% into MOPS-L containing different concentrations of Hb or Hn. In **F**, at t = 0 they were subcultured into BHI broth. The flasks were shaken at 37°C and absorbance at 600 nm (initially close to zero for all cultures) was monitored for 12–26 h (note different scales). Because of the slow growth of *L. monocytogenes* in iron-restricted minimal media, this graphic representation focuses on the comparison of the mutant strains at later times in the growth cycle.

**Figure 5:**
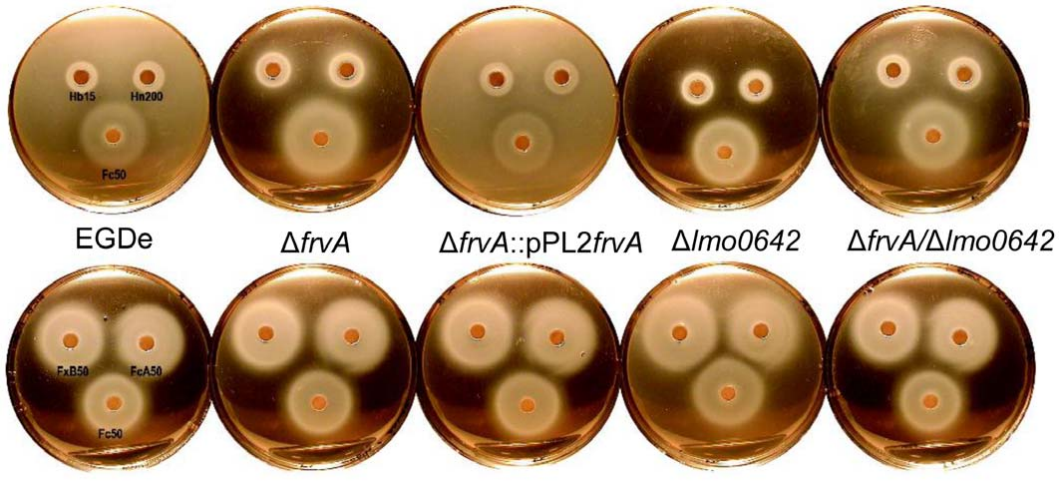
Nutrition tests. Tests demonstrate the halo of growth surrounding a paper disc embedded with 10 µl aliquots of the test iron compound. Concentrations of compounds are indicated as µM. Fc (ferrichrome) and FcA (ferrichrome A), FxB (ferrioxamine B), Hb (haem/haemoglobin) and Hn (haemin) were tested on BHI agar containing 0.1 mM BP. The experiment was repeated several times with similar results. No differences were seen between mutant strains and the wild-type in these iron nutrition assays.

During infection free iron is not available to bacterial cells whereas haem (Hb) and haemin (Hn) represent a potentially abundant source of iron [41]. However haem can be relatively toxic to cells at elevated concentrations [42], [43]. We investigated the rates of haemin uptake in Δ*frvA* and observed significant differences between the wild-type and mutant strains in the acquisition of [^59^Fe]-Hn (**Figure 3B**). The rate of haemin transport by Δ*frvA* (V_ma x_ = 30.6 pMol per 1×10^9^ cells per minute) was nearly twice that of the wild-type strain (Vmax = 18.8 pMol per 1×10^9^ cells per min). Subsequent analysis of the mutant in iron-limiting MOPS-L media supplemented with haemoglobin and haemin revealed that *L. monocytogenes* Δ*frvA* displayed growth behavior distinct from that of the wild-type and complement strains (**Figure 4**). Growth of the wild-type and complement was restored upon addition of increasing concentrations of Hb and Hn (0.2 and 2.0 µM) to iron-limiting media (**Figures 4A, 4C, 4F**). In contrast, growth of Δ*frvA* required addition of 0.2 µM Hn and Hb, whereas a higher concentration of 2.0 µM was shown to reduce growth suggestive of toxicity (**Figures 4B and 4E**). Nutrition tests were performed to assess the capability of the strains to utilize iron from several different sources. Δ*frvA* displayed no impairment in ability to utilize ferric siderophores, Hb or Hn when compared to the wild-type and complement strains (**Figure 5**).

As FrvA displays homology to bacterial heavy-metal transporting ATPases and with the knowledge that cation-transporting ATPases function in maintaining cation homeostasis [35], we investigated the sensitivity of Δ*frvA* to toxic levels of heavy metal sulfates such as copper, cobalt, cadmium, and zinc as well as iron. Exposure to a disk that contained 1 M FeSO_4_ resulted in a larger zone of clearance in Δ*frvA* when compared to the wild-type, indicative of elevated toxicity. However sensitivity to other heavy metals such as CdSO_4_, CoSO_4_, CuSO_4_ and ZnSO_4_ was comparable in both the wild-type and mutant (**Figure S3**). The data suggest that deletion of *frvA* does not affect the sensitivity of cells to heavy metals such as cadmium, cobalt, copper and zinc but confirms the contribution of this locus to iron homeostasis.

### Global disruption of iron homeostasis in the Δ*frvA* mutant

**Figure 6:**
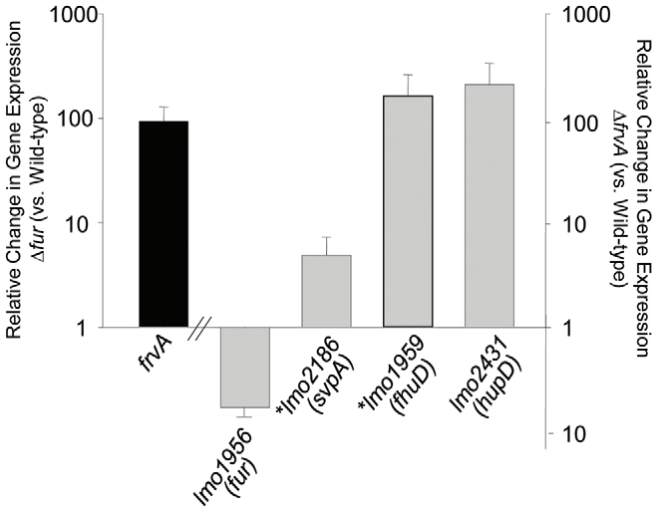
Quantitative real-time PCR. Induction of *lmo0641* (*frvA*) transcription in Δ*fur* compared to the wild-type (black bar) and induction of gene transcriptions in Δ*frvA* compared to the wild-type (gray bars) in BHI. Up-regulated genes are represented by bars above the x-axis and the down-regulated gene (*fur*) is represented by the bar below the axis. Asterisks represent Fur-regulated genes. Error bars represent the mean ± SD of the relative change in gene expression of independent duplicate samples.

As physiological analysis of *ΔfrvA* revealed iron-related phenotypes, we investigated the possibility that deletion of this locus could lead to altered expression of other genes in the *L. monocytogenes* genome involved in iron homeostasis. qRT-PCR was used to evaluate the differential expression of three iron-related genes in the wild-type and mutant strains (**Figure 6**). We chose two Fur-regulated genes; *lmo2186* which encodes a homologue of SaulsdC and bears homology to a haemin binding protein IsdC in *S. aureus* [44], and *lmo1959*, designated as *fhuD* encoding the *L. monocytogenes* ferrichrome binding protein [2], [44]. In addition, *lmo2431* (*hupD)* was also analyzed as this gene is part of the *hupDGC* operon encoding an ABC transporter involved in uptake of haemin and haemoglobin [2], [44]. qRT-PCR analysis revealed a strong induction of both *lmo2431* and *lmo1959* in *ΔfrvA* compared to the wild-type strain. *lmo2431* was shown to be up-regulated 210-fold and *lmo1959* up-regulated 164-fold in the mutant strain. *lmo2186* also displayed a an induction in *ΔfrvA*, with an almost 5-fold difference observed between the wild-type and mutant. As Fur is generally considered a repressor of transcription [16], the induction of two Fur-regulated genes in *ΔfrvA* is supported by our finding that the *fur* gene was shown to be down-regulated almost 6-fold in *ΔfrvA*.

### Conclusions

Using a functional genetics approach we identified a novel Fur-regulated locus (*frvA*) in *L. monocytogenes* that is essential for virulence and for resistance to haem and haemin-mediated toxicity. It is known that *L. monocytogenes* has the capacity to utilise iron-loaded haemoglobin and haemin as sources of iron [31]. Furthermore, elimination of haemoglobin and haemin uptake through mutation of the HupC transport system significantly impairs virulence potential, indicating that iron acquisition from haem is essential for pathogenesis [2]. However, haem and haemin are known to be toxic for bacteria and many bacteria express specific mechanisms for detoxification of haem [43].

FrvA possesses P-type ATPase and hydrolase conserved domains and is homologous to other heavy-metal transporting ATPases in *Staphylococcus* and *Bacillus*. Previous work by Francis and Thomas [35] identified another P-type ATPase, encoded by *ctpA*, which is involved in copper homeostasis in *L. monocytogenes*. Significantly, mutagenesis of the *ctpA* locus resulted in a strain that was unaffected in intracellular growth in the J774 macrophage cell line, but was impaired in ability to cause infection in the murine model [45]. Although P-type ATPases are known to mediate the transport of various heavy metals in bacteria, iron transport is most often associated with the structurally unique ATPases of the ABC transporter family [46]. However, Mta72, a P-type ATPase in *M. tuberculosis*, has been shown to transport iron transferred from the siderophore carboxymycobactin and is another rare example of a P-type ATPase involved in iron homeostasis [6].

It is interesting to note that the HrtA system in *S. aureus* also functions as a haem exporter and deletion of *hrtA* in that organism causes dysregulation of Fur expression resulting in pleiotrophic effects [47]. Whilst HrtA is an ABC transporter rather than a P-type ATPase we note homologies between FrvA and HrtA (21% identity over 221 amino acids). Certainly the results presented here suggest functional similarities between FrvA and HrtA though further experimental work will be necessary to directly compare both systems.

We did not demonstrate a role for FrvA in transport of ferric citrate or in iron reduction by *L. monocytogenes* and the mutant was not impaired in intracellular growth *in vitro*. Rather the predominant phenotype of Δ*frvA* is an increased uptake of haemin and significantly increased sensitivity to both haemin and haemoglobin toxicity and reduced virulence during systemic infection. However we acknowledge that further work is necessary to determine the precise biochemical mechanisms underpinning FrvA activity. The profound dysregulation of iron homeostasis in Δ*frvA* results in the de-repression of other Fur-regulated loci which complicates interpretation of the analysis of the mutant and which may necessitate the future use of isolated liposomal protein models to delineate its precise function.

## Materials and Methods

### Ethics statement

All animal procedures were approved by the University Research Ethics Board (UREB) in University College Cork (approval ID 2008/32) and were carried out in a specialized facility. Work was carried out under license from the Irish Department of Health.

### Bacterial strains, plasmids and culture conditions

*Listeria monocytogenes* strains were grown in Brain Heart Infusion (BHI) (Oxoid) broth at 37°C and *Escherichia coli* strains were grown in Luria-Bertani (LB) broth at 37°C. Strains and plasmids used in this study are listed in **Table 1**. For solid media, agar (1.5%) was added. Antibiotics, obtained from Sigma Chemical Company, were added in the following concentrations; 50 µg/ml ampicillin for pKSV7 in *E. coli* and 10 µg/ml chloramphenicol for pKSV7 in *L. monocytogenes*. For pPL2 in *E. coli* and *L. monocytogenes*, concentrations of 15 and 7.5 µg/ml chloramphenicol were used, respectively. Where indicated *L. monocytogenes* strains were sub-cultured at 1% into iron-deficient MOPS minimal salts medium [Neidhardt, 1974 #1524], with appropriate supplements (MOPS-L; [Xiao, #7571] to stationary phase (OD600 of approximately 1.2), and then subcultured again (1%) into MOPSL and grown to mid-log phase. Ferrichrome (Fc) and ferrichrome A (FcA) were purified from cultures of *Ustilago sphaerogena* [Emery, 1971 #2185]. Ferrioxamine B (FxB) was a gift from J. B. Neilands. We purchased purified hemin (Hn) and bovine hemoglobin (Hb) from Sigma-Aldrich (St. Louis, Mo).

**Table 1:** Bacterial strains and plasmids used in this study.

Strain or Plasmid	Relevant Properties	Reference
***E. coli***		
Top10	Chemically competent intermediate host, plasmid free	Invitrogen
***L. monocytogenes***		
EGDe	Wild-type strain, serotype 1/2a	W. Goebel
EGDpORI19::*2186*	EGDe derivative with an insertion into *lmo2186*	This study
EGDpORI19::*0365*	EGDe derivative with an insertion into *lmo0365*	This study
EGDpORI19::*2105*	EGDe derivative with an insertion into *lmo2105*	This study
EGDpORI19::*0641*	EGDe derivative with an insertion into *lmo0641*	This study
EGDpORI19::*1959*	EGDe derivative with an insertion into *lmo1959*	This study
EGDpORI19::*1960*	EGDe derivative with an insertion into *lmo1960*	This study
EGDpORI19::*0541*	EGDe derivative with an insertion into *lmo0541*	This study
EGDpORI19::*1131*	EGDe derivative with an insertion into *lmo1131*	This study
EGDpORI19::*2431*	EGDe derivative with an insertion into *lmo2431*	This study
EGDeΔ*fur*	EGDe derivative with *fur* deleted	[20]
Δ*frvA*	EGDe derivative with *lmo0641* deleted	This study
Δ*frvA*pPL2::*frvA*	Δ*frvA* with pPL2-frvA integrated on the chromosome	This study
	at the tRNAArg-attB′ site	
Δf*rvA*[85–416]	EGDe derivative with a central portion of *lmo0641*	This study
	deleted (from residue 85–416)	
Δ*frvA*[85–416]pPL2::*frvA*	Δ*frvA* with pPL2-*frvA* integrated on the chromosome	This study
	at the tRNAArg-attB′ site	
Δ*lmo0642*	Deletion mutant in *lmo0642*	This study
**Plasmids**		
pKSV7	Cm^R^ , Temperature Sensitive	[51]
pPL2	Cm^R^, Integrates on the chromosome at the PSA phage	[52]
	attachment site within the tRNAArg gene	

Cm^R^, chloramphenicol resistant.

### DNA manipulations

Gel extraction was performed using the Qiagen Gel Extraction Kit (Qiagen). Plasmid DNA isolation was carried out utilizing the Qiagen QIAprep Spin Miniprep Kit (Qiagen). PCR reagents and T4 DNA ligase, supplied by Roche Diagnostics GmbH (Mannheim, Germany), and restriction enzymes (New England Biolabs) were all used according to the manufacturer's instructions. Oligonucleotide primers were synthesized by MWG and are listed in **Table S2**. PCR reactions were completed using a PTC-200 (MJ Research) PCR system. Colony PCR was performed following lysis of cells with IGPAL CA-630 (Sigma). Genomic DNA was isolated from *L. monocytogenes* using a chromosomal kit (Sigma) according to the manufacturer's instructions.

### Creation of plasmid insertion mutants

A central portion of the gene of interest was amplified by PCR and cloned into the multiple cloning site of pORI19 (RepA^−^) [20], [28]. Following plasmid isolation, electrotransformation of *L. monocytogenes* EGDe containing pVE6007 (RepA^+^/Temperature sensitive) was performed according to the protocols outlined by Park and Stewart, (1990). Loss of pVE6007 was achieved by transferring 10 µl of a 30°C overnight culture to BHI broth prewarmed to 42°C with subsequent growth for 16 hrs at 42°C and isolation on prewarmed BHI-Em (5 µg/ml) agar plates at 42°C. Loss of pVE6007 (Cm^s^) was confirmed by replica plating onto BHI-Em (5 µg/ml) and BHI-Cm (10 µg/ml) plates with overnight incubation at 30°C. Integration results in the formation of a stable Em^r^ mutant and was confirmed by PCR using a primer outside the region of integration and a primer specific to the plasmid.

### Construction of deletion mutants

As described by Horton *et al*. [48] the Splicing by Overlap Extension (SOE) procedure was utilized to create a complete gene deletion mutant. This is an in-frame, non-polar deletion of a gene in the *L. monocytogenes* EGDe chromosome. Two pairs of primers were designed, SOEA/SOEB and SOEC/SOED, to amplify two fragments of approximately equal size on either side of the gene to be deleted using the proofreading enzyme Vent polymerase (New England Biolabs). These AB and CD products were then gel extracted to ensure purity, mixed in a 1∶1 ratio, and were spliced together using SOEA/SOED primers. This AD product was digested and cloned into pKSV7, a temperature sensitive plasmid. The resulting construct was electroporated into competent *E. coli* DH5α cells and transformants were selected on Luria-Berani plates with ampicillin. The plasmid was isolated using the Qiagen QIAprep Spin Miniprep kit. The presence of the correct insert was verified by sequencing (Lark Technologies Inc., Essex, UK) using the pKSV7 MCS primers M13F and M13Rmut. The isolated plasmid was electroporated into competent *L. monocytogenes* EGDe cells. Transformant selection took place on Brain-Heart Infusion agar containing chloramphenicol. Clones in which chromosomal integration of the plasmid had occurred are selected by serial passaging at 42°C and are streaked onto pre-warmed BHI-Cm agar plates. Continuous passaging at 30°C in BHI broth followed by replica plating onto BHI and BHI-Cm plates ensures plasmid excision. Chloramphenicol sensitive colonies were tested for gene deletion using primers SOEX, located upstream, and SOEY, located downstream of the gene of interest.

### Complementation of deletion mutants

A site-specific phage integration vector, pPL2, was used for the complementation of SOEing deletion mutants. This vector integrates within the tRNA^Arg^ gene on the chromosome. Vent polymerase (New England Biolabs), a proof reading enzyme, was used to amplify the entire deleted gene and flanking regions, including the upstream gene promoter. Primers CompA and CompB included restriction sites corresponding to those on the MCS site of pPL2. The PCR product was gel extracted to ensure purity, and was digested and cloned into pPL2. The resulting construct was electroporated into competent *E. coli DH5α* cells and transformants were selected on Luria-Berani plates with chloramphenicol. The plasmid was isolated using the Qiagen QIAprep Spin Miniprep kit. The presence of the correct insert was verified by sequencing (Lark Technologies Inc., Essex, UK) using the pPL2 MCS primers T3F and T7R. The isolated plasmid was electroporated into competent SOEing mutant cells. Transformant selection took place on Brain-Heart Infusion agar containing chloramphenicol. The presence of the gene was authenticated using a forward running check primer that anneals to the middle of the gene and SOE D, located on the cloned insert. Integration of pPL2 to the correct site was confirmed using primers PL102, located upstream of the integration site, and the SOE D primer.

### Bioinformatics

Nucleotide and amino acid sequences of Listerial genes and proteins were obtained from the Listilist website at http://genolist.pasteur.fr/listilist/. ExPASy proteomics tools webside at http://www.expasy.ch/tools/ was used for protein-related bioinformatics. This site included links to: NCBI (http://www.ncbi.nlm.nih.gov/blast/Blast.cgi) for blasting, ScanProsite (http://www.expasy.ch/tools/scanprosite/) for motif searching, SOSUI (http://bp.nuap.nagoya-u.ac.jp/sosui/) for transmembrane region predictions, TMHMM (http://www.cbs.dtu.dk/services/TMHMM-2.0/) for TM helixes, PredictProtein (http://www.predictprotein.org/) for TM helix location and topology, and TMpred (http://www.ch.embnet.org/software/TMPRED_form.html) for protein orientation. The post-genome database for *Listeria* Research (http://leger2.gbf.de/cgi-bin/expLeger.pl) was utilized for gene functions and subcellular localization of proteins.

### RNA extraction

Total RNA was extracted using both the Macaloid Clay method, outlined by Raya *et al*. [49], and the Qiagen RNeasy Mini Kit. Cultures were grown overnight shaking at 37°C. A 1% inoculum was added to 30 mLs BHI broth and cultures were grown at 37°C until an OD_600 nm_ of 0.3 was reached. 30 mLs of culture were pelleted by centrifugation at 4,000 g for 7 minutes. The supernatant was removed, the pellet was washed with 1 mL cold TE buffer (10 mM Tris, 1 mM EDTA: pH 8.0), and centrifuged again for 13,000 g for 1 minute. Again the supernatant was removed, and the pellet was resuspended in 20 µL lysozyme (50 mg/mL), 400 µL cold TE buffer, and left at room temperature for 3 minutes. Subsequently, the cell suspension was added to a 1.5 mL screw-cap plastic tube containing 50 µL 10% sodium dodecyl sulphate, 500 µL phenol-chloroform (5∶1), 175 µL Macaloid Clay and 0.5 g 425–600 µm glass beads (Sigma). Cell disruption was achieved using a bead beater (Mini-beadbeater 8TM cell disrupter, Biospec products.) Cells were beaten for 1 minute, placed on ice for 1 minute, beaten again for 1 minute, and then centrifuged for at 13,000 g for 3 minutes. The organic layer was removed and precipitated with 1∶10 volume sodium acetate, and 2.5 volume 96% ethanol at −80°C for 20 minutes. Following this step, samples were put through the Qiagen RNeasy Mini Kit and then eluted in 50 µL TE buffer. RNA samples were treated with RNase-free DNase I set (Qiagen) and DNA-free (Ambion) was used to remove any DNA. The concentration of RNA was quantified utilizing a Nano-Drop (ND-1000 spectrophotometer). A PCR, carried out with 16S rRNA primers; L142 and U141, was used to ensure the absence of DNA in the samples. The reverse transcriptase PCR was run using 4 µL random primer p(dN)_6_, 2 µL RNA, and 2 µL DEPC water (Sigma) at 65°C for 10 min, and put directly on ice. To these samples, 32 µL of a mastermix was added containing; 1 µL Expand Reverse Transcriptase (Roche), 8 µL 5× Buffer (Roche), 4 µL 100 mM dTT (Roche), 1 µL dNTP mix (dATP, dCTP, dGTP, dTTP; 10 mM) and 18 µL DEPC water. This reaction was carried out at 30°C for 10 min, 42°C for 3 hours, and held at 4°C. cDNA was confirmed through PCR using L142 and U141 primers and the wild-type *L. monocytogenes* extracted DNA as a positive control.

### Quantitative real-time PCR

The Universal Probe Library Assay Design Center (https://www.roche-applied-science.com/sis/rtpcr/upl/adc.jsp) was used to design PCR primers which correspond to a specific probe in the library. Primer sequences and corresponding probes are listed in **Table S2**. The 16S rRNA gene was used as a housekeeping gene to compensate for any variability in the initial amount of starting total RNA. Amplification reactions consisted of 2.5 µL of cDNA, 6.4 µL of 2× FastStart TaqMan Probe Master (Roche), primers (900 nM) and probe mix (250 nM). RNase-free water was added to bring the total volume of the reaction to 10 µL. Reactions were performed in duplicate on 384-well plates using the LightCycler 480 System (Roche). Negative control reactions, without cDNA, were also included on the plate. Thermal cycling conditions were carried out according to manufacturer's instructions (Roche) and the 2^−ΔΔCt^ method [50] was used to calculate the relative changes in gene expression from the qRT-PCR experiments.

### Growth curves

Growth of *Listeria monocytogenes* in MOPS-L media. EGD-e and its mutant derivatives were grown in BHI overnight, and then subcultured at 1% into BHI broth or MOPS-L media. In the latter case, the bacteria were grown to stationary phase, and for growth rate determinations they were subcultured again at 1% into MOPS-L containing Hn or Hb at varying concentrations. The cultures were shaken at 37°C and OD_600 _nm was monitored at indicated time points up to 26 hours.

### Metal disk assay

Cultures were grown overnight shaking at 37°C. A 2% inoculum was added to 10 mL of fresh BHI and cultures were grown to logarithmic phase (0.3OD) at 37°C. 400 µL of log phase cell cultures were added to 4 mL of cooled, molten soft agar (0.75%) and poured on top of a petri dish containing 20 mL BHI agar. After solidifying, a sterilized 13 mm disk (Whatman) was placed on top of the overlay. Metals used were made up in 1 M stocks in which 35 µL of each metal were dispensed onto the center of the disk. The plate was then incubated overnight at 37°C, and the zone of clearance surrounding the disk was measured.

### ^59^Fe binding and uptake experiments

For binding and transport determinations, we prepared ^59^Fe complexes of citrate (specific activity 150 to 1,000 cpm/pMol) and haemin [44]. For ^59^Fe-citrate, we provided the organic ligand in 50-fold molar excess. We conducted adsorption and transport experiments [2], [44] over a range of concentrations, by adding appropriate amounts of ^59^Fe complexes to two aliquots of 2×10^7^ cells of EGD-e or its mutants, and incubating the aliquots for 15 s and 75 s, respectively, before collecting and washing the cells on 0.2 micron filters. The 15 s aliquot measured the amount initially bound to the cells, which when subtracted from the second time-point, gave the amount transported during a 1 min period. At each concentration, data were collected in triplicate and averaged. The K_d_ and capacity of ^59^Fe-siderophore binding were determined by using the “Bound-versus-Total” equation of Grafit 5.09 (Erithacus, Ltd., Middlesex, UK), and K_m_ and V_max_ of transport were calculated using the “Enzyme Kinetics” equation.

### Macrophage assay

This intracellular survival assay was carried out using J774 mouse macrophage cells (originally obtained from the American Type Culture Collection, Manassas, VA). 24-well tissue culture plates were seeded with 1×10^5^ live cells per well in DMEM (Gibco) containing 10% fetal calf serum and incubated in 5% CO_2_ at 37°C for 40 hours. For infection, bacteria were prepared by centrifuging 1 mL of an overnight culture which was then washed once in PBS, and resuspended in 1 mL DMEM. Bacteria were diluted in DMEM and 1×10^7^ CFU was added to each well containing washed macrophage cells. To increase contact between macrophages and bacteria, the 24-well plates were centrifuged at 1500 rpm for 10 min and incubated for 1 hour in 5% CO_2_ at 37°C. To kill extracellular bacteria, 1 mL of 100 µg/mL gentamycin (Sigma) was added to each well and incubated for an additional 30 min. Bacteria surviving intracellularly were enumerated at time points taken after addition of gentamycin. Each well was washed twice in PBS, cells were lysed with 250 µL ice cold water containing 0.02% Triton X (Sigma), and scraped in a similar manner using a pipette tip. Serial dilutions were carried out on the lysate and plated on BHI agar overnight at 37°C.

### Murine virulence assay

Cultures were grown overnight with shaking at 37°C. Cultures were centrifuged, washed in PBS (Sigma), resuspended and diluted to 1×10^6^ CFU/mL in PBS. BALB/c mice were inoculated with 4×10^5^ CFU in 200 µL PBS intraperitoneally (i.p.). The mice were euthanized 3 days post-infection. Spleens and Livers were harvested and then homogenized in PBS. Bacteria were enumerated by plating the serial dilutions of organ homogenates on BHI agar left to incubate overnight at 37°C.

### *Galleria mellonella* virulence assay

Cultures were grown overnight with shaking at 37°C. Cultures were centrifuged, washed, and resuspended in an equal volume of PBS (Sigma). Infection of *Galleria mellonella* was performed according to the protocol outlined by Joyce *et al*. [32]. Briefly, insects were obtained from Livefood, UK and were stored in the dark at room temperature prior to use. 3 groups, containing 10 insects per group, were injected with 1×10^6^ CFU/10 µL of the wild-type *L. monocytogenes* EGD-e strain (group 1), 1×10^6^ CFU/10 µL EGDeΔ*0641* (group 2), or10 µL PBS (group 3) to serve as a negative control. Bacterial suspensions were injected using a sterile Hamilton syringe and a 30-Gauge disposable needle into the first right pro-leg of the second set of pro-legs. All ten insects per group were placed together in a Petri-dish lined with Whatman paper and incubated in the dark at 37°C. Insects were examined over several days and time of death was recorded.

## Supporting Information

Figure S1 (A) Further confirmation of a role for *lmo0641* (*frvA*) in virulence using an in-frame deletion mutant Δ*frvA*_[85–416]_ and complemented strain Δ*frvA*_[85–416]_::pPL2*frvA*. Mice were injected i.p. with the appropriate strains and the number of bacteria recovered from the spleen was determined three days post-infection. (B) The ability of Δ*frvA*_[85–416]_ mutants (○) to survive in vivo in comparsion to the wild-type (•) was assessed over three days. Numbers in the spleens of infected animals was determined daily. Error bars represent the standard deviations from the mean (n = 4).
(PPTX)

Figure S2 Invasion and intracellular growth of mutant and wild-type strains in the J774 macrophage cell line. Error bars represent standard deviations of triplicate experiments. Students t-test did not indicate significant differences between groups at any time points.
(PPTX)

Figure S3 Metal toxicity disk assay. 35 µL of 1 M copper, cobalt, cadmium and zinc sulfates (A) or iron sulfate (B) were added to a 13 mm disk placed on an overlay of wild-type or Δ*0641* cells grown up to 0.3 OD. Plates were incubated for 24 over night and zones of clearance (ZOC) were measured (mm). No statistical differences were observed between strains in the sensitivity to heavy metals CdSO4, CoSO4, CuSO4 and ZnSO4. While no ZOC was observed around disks containing 1 M FeSO4 for the wild-type, a ZOC of 7.5 mm±0.5 mm was seen for Δ*frvA*. Experiments were done in triplicate.
(PPTX)

Table S1 **Iron Reductase Assays.** The listerial strains were grown in chemically defined media (CDM) as described by Premaratne *et al.*, (1991) at 37°C at 200 rpm until the cells reached approximately 75% of their maximum growth. The supernatant fluids were harvested by centrifugation using a microfuge and stored at −80°C until assayed for reductase activity and protein. Iron reductase activity was carried out by reacting the culture supernatant fluids with Fe^3+^-NTA (nitrilotriacetic acid), 1∶5, at a final iron concentration of 5×10^−5^ M, in 25 mM Tris-HCl, pH 7.4, containing BPS (bathophenanthroline sulphonate – Sigma Chemical Company) at a final concentration of 2.5×10^−4^ M. The reaction was followed at 535 nm in a Cary 50 spectrophotometer and the initial velocities were determined. The control consisted of uninoculated media that was treated in the same manner as the listerial strains. The nonspecific reduction of iron by the uninoculated media (8.80×10^−10^ Ms^−1^) was subtracted from each test value. The supernatant fluids were assayed for their protein concentration (BioRad) and the values were reported as the initial velocity of the reduction of iron (Vi), in Mol/sec/µg protein.
(DOCX)

Table S2 **Oligonucleotide primers used in this study.**
(DOCX)
