# Supplementary figures and images for: A Putative P-Type ATPase Required for Virulence and Resistance to Haem Toxicity in Listeria monocytogenes
Source: PLoS One. 2012 Feb 21;7(2):e30928. doi: 10.1371/journal.pone.0030928 (PMC3283593; doi:10.1371/journal.pone.0030928)

## Slide 1
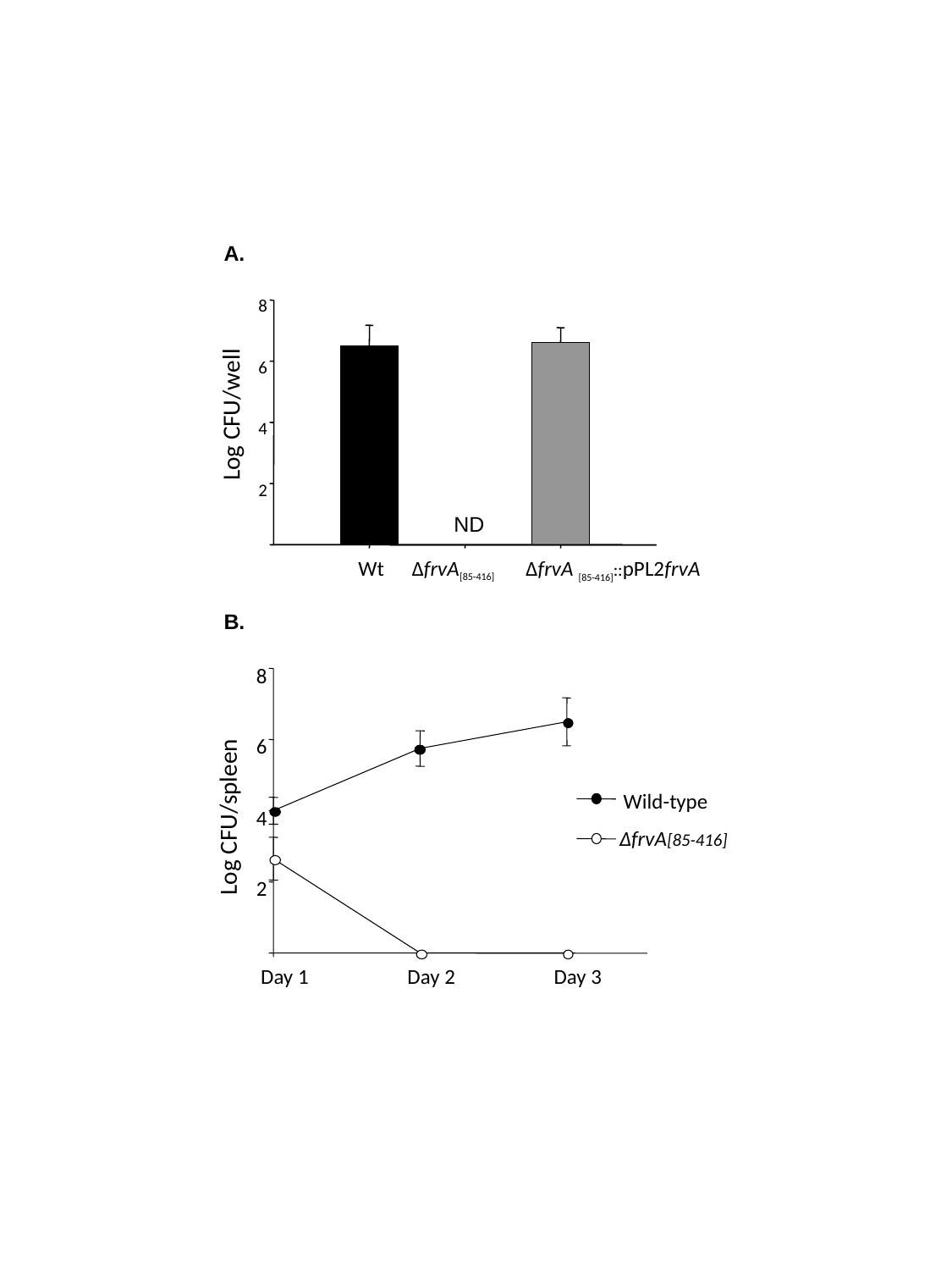

A.
8
6
Log CFU/well
4
2
ND
Wt
ΔfrvA[85-416]
ΔfrvA [85-416]::pPL2frvA
B.
8
6
4
2
Day 1
Day 2
Day 3
Wild-type
Log CFU/spleen
ΔfrvA[85-416]

Supplement: Figure S1 — (A) Further confirmation of a role for lmo0641 (frvA) in virulence using an in-frame deletion mutant ΔfrvA [85–416] and complemented strain ΔfrvA [85–416]::pPL2frvA. Mice were injected i.p. with the appropriate strains and the number of bacteria recovered from the spleen was determined three days post-infection. (B) The ability of ΔfrvA [85–416] mutants (○) to survive in vivo in comparsion to the wild-type (•) was assessed over three days. Numbers in the spleens of infected animals was determined daily. Error bars represent the standard deviations from the mean (n = 4). (PPTX) [file pone.0030928.s001.pptx]

## Slide 1
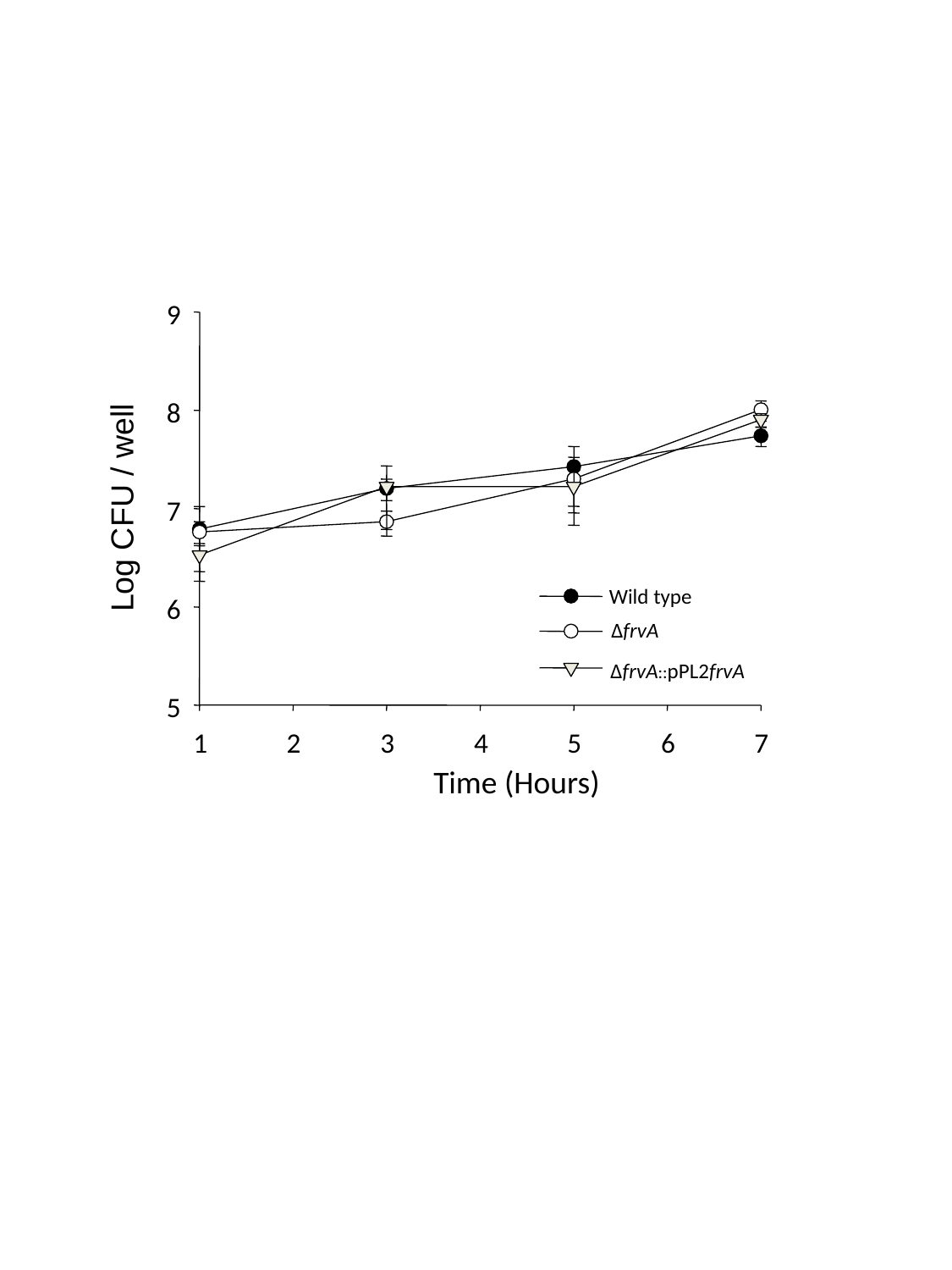

9
8
7
6
5
1
2
3
4
5
6
7
Time (Hours)
Log CFU / well
 Wild type
ΔfrvA
ΔfrvA::pPL2frvA

Supplement: Figure S2 — Invasion and intracellular growth of mutant and wild-type strains in the J774 macrophage cell line. Error bars represent standard deviations of triplicate experiments. Students t-test did not indicate significant differences between groups at any time points. (PPTX) [file pone.0030928.s002.pptx]

## Slide 1
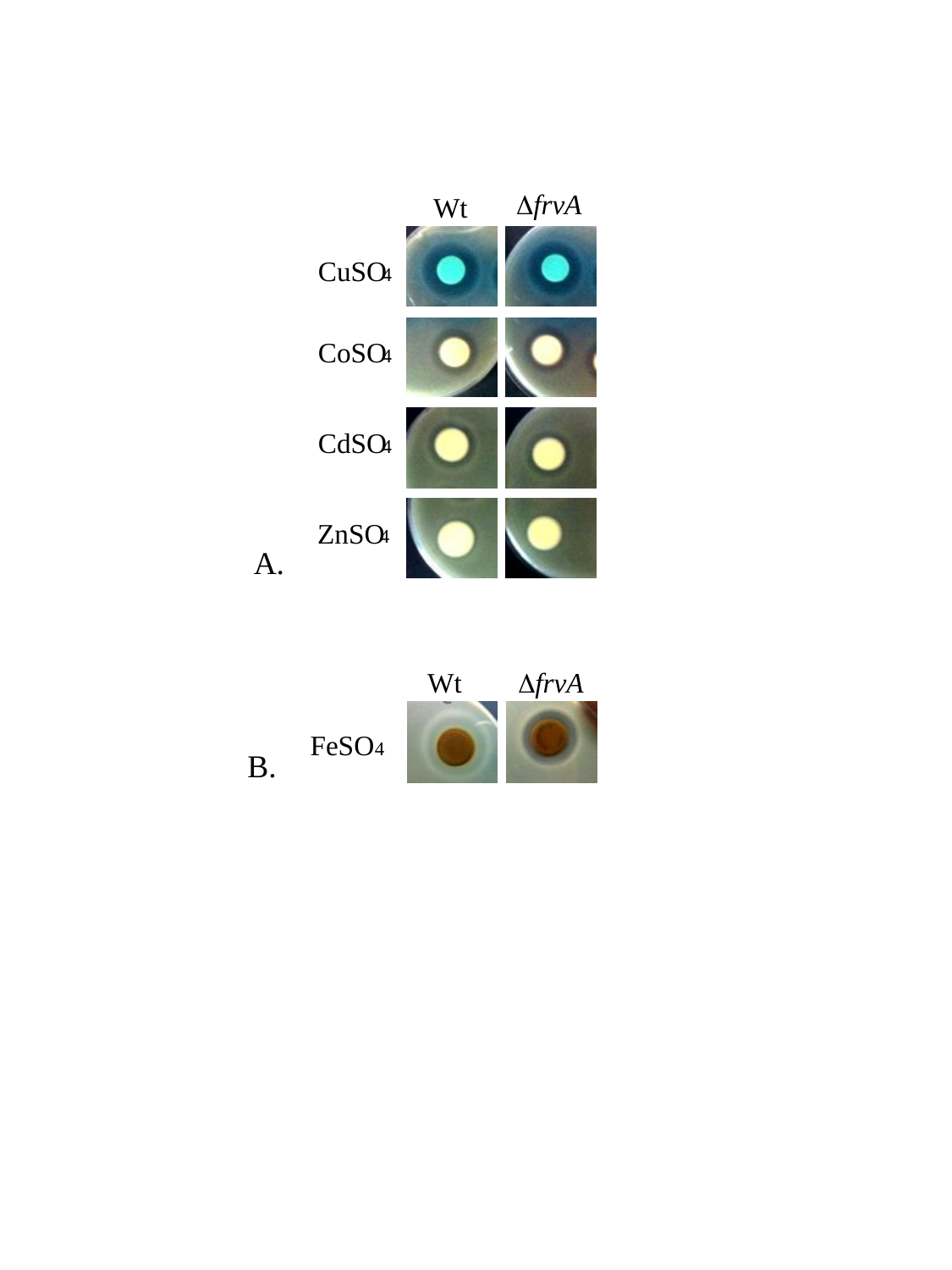

Wt
CuSO
4
CoSO
4
CdSO
4
ZnSO
4
A.
DfrvA
Wt
DfrvA
FeSO
4
B
.

Supplement: Figure S3 — Metal toxicity disk assay. 35 µL of 1 M copper, cobalt, cadmium and zinc sulfates (A) or iron sulfate (B) were added to a 13 mm disk placed on an overlay of wild-type or Δ0641 cells grown up to 0.3 OD. Plates were incubated for 24 over night and zones of clearance (ZOC) were measured (mm). No statistical differences were observed between strains in the sensitivity to heavy metals CdSO4, CoSO4, CuSO4 and ZnSO4. While no ZOC was observed around disks containing 1 M FeSO4 for the wild-type, a ZOC of 7.5 mm±0.5 mm was seen for ΔfrvA. Experiments were done in triplicate. (PPTX) [file pone.0030928.s003.pptx]
