# Supplementary material for: A Putative P-Type ATPase Required for Virulence and Resistance to Haem Toxicity in Listeria monocytogenes
Source: PLoS One. 2012 Feb 21;7(2):e30928. doi: 10.1371/journal.pone.0030928 (PMC3283593; doi:10.1371/journal.pone.0030928)
Supplement: Table S1 — Iron Reductase Assays. The listerial strains were grown in chemically defined media (CDM) as described by Premaratne et al., (1991) at 37°C at 200 rpm until the cells reached approximately 75% of their maximum growth. The supernatant fluids were harvested by centrifugation using a microfuge and stored at −80°C until assayed for reductase activity and protein. Iron reductase activity was carried out by reacting the culture supernatant fluids with Fe3+-NTA (nitrilotriacetic acid), 1∶5, at a final iron concentration of 5×10^−5^ M, in 25 mM Tris-HCl, pH 7.4, containing BPS (bathophenanthroline sulphonate – Sigma Chemical Company) at a final concentration of 2.5×10^−4^ M. The reaction was followed at 535 nm in a Cary 50 spectrophotometer and the initial velocities were determined. The control consisted of uninoculated media that was treated in the same manner as the listerial strains. The nonspecific reduction of iron by the uninoculated media (8.80×10^−10^ Ms−1) was subtracted from each test value. The supernatant fluids were assayed for their protein concentration (BioRad) and the values were reported as the initial velocity of the reduction of iron (Vi), in Mol/sec/µg protein. (DOCX) [file pone.0030928.s004.docx]

Table S1. Iron Reductase Assay

| Strain | Vi; Mol/sec/μg protein |
| --- | --- |
| WT  Δ*frvA*_[85-416]_  Δ*frvA*_[85-416]_*::pPL2frvA* | 3.19 X 10^-9^  2.67 X 10^-9^  1.20 X 10^-9^ |
