# Supplementary material for: A Putative P-Type ATPase Required for Virulence and Resistance to Haem Toxicity in Listeria monocytogenes
Source: PLoS One. 2012 Feb 21;7(2):e30928. doi: 10.1371/journal.pone.0030928 (PMC3283593; doi:10.1371/journal.pone.0030928)
Supplement: Table S2 — Oligonucleotide primers used in this study. (DOCX) [file pone.0030928.s005.docx]

**Table S2**. Oligonucleotide primers used in this study

| **Primer** | **Sequence** | **qRT-PCR Probe** |
| --- | --- | --- |
| 2186F | TGATAAAGCTTCAACGGTAAC |  |
| 2186R | GCTAATGCTTTTCTAGATCC |  |
| 0365F | CGCAAATTCTAGATTTAAAAACA |  |
| 0365R | TGCTTGGTTAAGCTTACCTTC |  |
| 0641F | ATACGATCTGCAGTAAAATTATTCG |  |
| 0641R | CCAATTCCCTTCTAGATAAATCGC |  |
| 1960F | ACCACAAAAGCTTTCTGCACC |  |
| 1960R | TGGTGAGCGTATCTAGATGCG |  |
| 1959F | TGGAATGAAGCTTGTGGGGGCCA |  |
| 1959R | TAAATCTCTAGACCACCGCGC |  |
| 0541F | GGACAGAAAGCTTTGGTAATGAC |  |
| 0541R | GGAATTGCTTCTCTAGAAATAGC |  |
| 1131F | GAATTTAGGATCCAAATTCCAT |  |
| 1131R | CTATTTGCATCTAGATAACC |  |
| 2105F | GTTTGACTGCAGTCGAATACACG |  |
| 2105R | AGTGATGGTCGACAAGCGC |  |
| 2431F | AGAAAGCTGCAGAAGTCGGCA |  |
| 2431R | CCGTGAATCATCTAGAAAATC |  |
| 1007F | ACCTGGTAAATACGAAGCTC |  |
| 1007R | TTCGATTAGAAGTAGCGGTT |  |
| 0484F | AGAAAAAGGCGCAGCAGAGC |  |
| 0484R | AAGAGAAGACCGCAAAGGCA |  |
| 2186int | GGGAGATTTAAGAATGAAGA |  |
| 0365int | GACCTATCAAGATGGTACAT |  |
| 0641int | GGCTCAACCACATTAAATGA |  |
| 1960int | AACCGCTTTAGTAGGTGCAA |  |
| 1959int | ACATATACAATGGCAAATGG |  |
| 0541int | AACGCCGAAAAGAATTGTCG |  |
| 1131int | GGAAGTTTAGTTGGATTTGC |  |
| 2105int | AACGACTAGCCCTATGTTTG |  |
| 2431int | AACCAGAACGAATTATCGCA |  |
| *frvA-*SOE A | GCG**GAATTC**GTCAAGGATTCTT |  |
| *frvA-*SOE B | CTTAGACTAGGAATAGACAAGCAGTGAAATTCACT |  |
|  | ATCAGTCTAATACACA |  |
| *frvA-*SOE C | CTGCTTGTCTATTCCTAGTCTAAG |  |
| *frvA-*SOE D | CAT**TCTAGA**GTTGGCGATTTTGTGAAC |  |
| *frvA-*SOE X | TTGAAACGAATAACAATTGG |  |
| *frvA-*SOE Y | CCCGTATCTAAAAACATTTCC |  |
| *frvA-*COMP F | CGC**GGATCC**CCAGGAAGAATTGCTGATATT |  |
| *frvA-*COMP R | AAAA**CTGCAG**CCCGTATCTAAAAACATTTCC |  |
| *frvA*_[85-416]_-SOEA | GAGAGGATCCGCATAATGAAA |  |
| *frvA*_[85-416]_-SOEB | CCAATAACCAATAATCGATGC |  |
| *frvA*_[85-416]_-SOEC | GCATCGATTATTGGTTATTGGTCAAATGGCGCATT |  |
|  | TGAACGA |  |
| *frvA*_[85-416]_-SOED | CAATTCTAGACACATCCAC |  |
| MI3F | GTTTTCCCAGTCACGAC |  |
| M13Rmut | CAGGAAACACGTATGAC |  |
| T3F | AATTAACCCTCACTAAAGG |  |
| T7R | TAATACGACTACTATAGGG |  |
| L142 | GAGTGCTTAATGCGTTAG |  |
| U142 | TTGCTCTTCCAATGTTAG |  |
| *16S rRNA* F | GAAAGCCACGGCTAACTACG | 66 |
| *16S rRNA* R | GACAACGCTTGCCACCTAC | 66 |
| *0641* F | AAAACTGTTGGTGCGGATGT | 31 |
| *0641* R | TCGTATTTTCAAATGTTTGTGTTACTT | 31 |
| *2186* F | TTTCGATGAAGGATCTGCAA | 78 |
| *2186* R | TGTTGCGGGTGTTGTTGTAT | 78 |
| *2431* F | CAACAAGCGCCGTTAAAAAT | 11 |
| *2431* R | CAGGATTGGTTCCGAAAAGT | 11 |
| *1959* F | TACGCCAAATCGCTGACTTA | 19 |
| *1959* R | TTGATGAAACTTATCTAGCCATGC | 19 |

Restriction sites are in boldface and complementary overhangs are underlined
